# Host-HIV-1 Interactome: A Quest for Novel Therapeutic Intervention

**DOI:** 10.3390/cells8101155

**Published:** 2019-09-27

**Authors:** Ekta Shukla, Radha Chauhan

**Affiliations:** National Center for Cell Science, S.P Pune University, Pune-411007, Maharashtra, India; ektashukla19@gmail.com

**Keywords:** anti-retroviral therapy, drug resistance, protein-protein interactions, host factors, nuclear pore complex

## Abstract

The complex nature and structure of the human immunodeficiency virus has rendered the cure for HIV infections elusive. The advances in antiretroviral treatment regimes and the development of highly advanced anti-retroviral therapy, which primarily targets the HIV enzymes, have dramatically changed the face of the HIV epidemic worldwide. Despite this remarkable progress, patients treated with these drugs often witness inadequate efficacy, compound toxicity and non-HIV complications. Considering the limited inventory of druggable HIV proteins and their susceptibility to develop drug resistance, recent attempts are focussed on targeting HIV-host interactomes that are essential for viral reproduction. Noticeably, unlike other viruses, HIV subverts the host nuclear pore complex to enter into and exit through the nucleus. Emerging evidence suggests a crucial role of interactions between HIV-1 proteins and host nucleoporins that underlie the import of the pre-integration complex into the nucleus and export of viral RNAs into the cytoplasm during viral replication. Nevertheless, the interaction of HIV-1 with nucleoporins has been poorly described and the role of nucleoporins during nucleocytoplasmic transport of HIV-1 still remains unclear. In this review, we highlight the advances and challenges in developing a more effective antiviral arsenal by exploring critical host-HIV interactions with a special focus on nuclear pore complex (NPC) and nucleoporins.

## 1. Introduction

HIV (Human Immunodeficiency Virus), a major killer worldwide was first recognised in the early 1980s in the United States as a strange retrovirus causing an entirely new disease called AIDS (Acquired Immunodeficiency Syndrome) [1]. To date, we do not have any cure for AIDS. The current anti-HIV treatment (Anti-Retroviral Therapy, or ART) has certainly resulted in a dramatic decrease in AIDS and AIDS-related complications in treated patients; however, the emergence of drug resistance and side effects posed by conventional medicines are currently restraining the clinical use of many of these antiviral compounds. In this review, we emphasize on the significance of host-viral interactions, particularly HIV-1 and human interactomes, which are now being revisited for exploring novel drug targets with higher efficacy. Furthermore, focus has been laid on the HIV-1 dependence on different host factors in the nuclear import and export of viral nucleic acids and proteins. While proper nuclear entry and exit is essential for viral replication, the nuclear pore complex can be a key target for virus subversion and may provide the basis for developing antiviral compounds for therapeutic intervention that not only target viral proteins alone but their interactions with nuclear membrane partners like nucleoporins. We also highlight the current challenges and further scope in the field. The lack of complete molecular understanding and structural knowledge of the host-viral protein complexes is the most significant limiting factor in the area.

### Global Burden and Geographic Distribution of HIV

**Figure 1:**
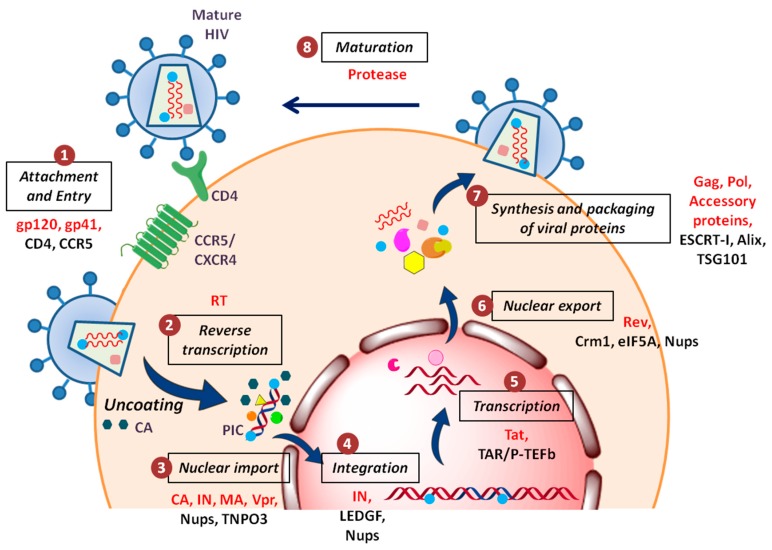
Schematic overview of the HIV-1 replication cycle: (1) The HIV-1 particle attaches and fuses itself to the host cell membrane via its surface glycoproteins and enters the cell cytoplasm; (2) viral genomic RNA is reverse transcribed into DNA, accompanied by uncoating of the viral capsid; (3) nuclear entry of pre-integration complex occurs with the help of various host nuclear proteins; (4) integration of vDNA into the host chromatin; (5) during productive infection, viral transcription takes place; (6) RNA splicing and nuclear export of viral RNA occurs; (7) production and assembly of new virus particles, which bud from the plasma membrane; and (8) virions become infectious after maturation (action of protease). The viral and host cellular proteins involved in the replication cycle of HIV-1 are highlighted at each step in red and black, respectively.

HIV arose through zoonotic transmissions of simian immunodeficiency viruses (SIV) during the past century in East and Central Africa [2,3]. Today, HIV-1, along with its less widespread cousin HIV-2, is responsible for a worldwide pandemic, infecting more than 36 million people. On the other hand, HIV-2 is relatively less prevalent and restricted to West Africa and some parts of Asia. Moreover, the infection with HIV-2 seems to be slowly progressing and less efficiently transmissible. These retroviruses are divided into different groups based on their genetic diversity. While HIV-2 has five groups and no subtypes/clades, three groups of HIV-1 have been identified, viz. M, N, and O, based on their genome differences. Most of the HIV-1 infections are caused by group M viruses which are further classified into nine clades (A–D, F–H, J and K) [4,5]. The DNA sequences of viruses belonging to distinct clades differ by 15–20%. Apparently, the extent of genetic divergence among the subtypes account for the different levels of their pathogenic efficiency and potential. Therefore, the evolutionary success of these viruses lies in their ‘deceptive simplicity’, i.e., even though these viruses possess a small genome and encodes few similar proteins, they are difficult to target due to their enormous genetic variations and capability of mutation. Despite encoding only 15 mature proteins (Table 1), HIV-1 can persistently attack and compromise the innate and adaptive immune systems of an individual [6]. Furthermore, for a successful viral replication, it employs a series of well-planned steps which engage complex relationship between cellular and viral factors (Figure 1).

One of the surprising facets of the HIV/AIDS pandemic is the unequal distribution of HIV. In the year 2018, an estimated 37.9 million individuals were living with HIV worldwide with highest burden witnessed in southern and eastern Africa. In the same year, an estimated 1.7 million new HIV cases were reported [7]. Every year, the number of deaths occurring globally from HIV-related causes reaches 1.0 million. Even though no population or continent has been spared by HIV, some regions and populations have been affected far more than the others [8]. This indicates that a varied combination of biological and social factors governs the spread of HIV. Moreover, human susceptibility to HIV infection also differs. Vulnerability to infection depends on multiple aspects like genetic factors, innate resistance and psychosocial determinants of health, such as poverty, stigma, gender-based violence, mental health, etc. 

## 2. HIV-1 Antiretroviral Therapy: Current Status and Challenges 

ART mainly targets the enzymatic processes of the HIV-1 replication cycle. The current conventional antiretroviral drugs are classified into six classes of therapeutic mechanisms (Table 2) [25,26,27]. However, soon after the success of the first drug, Azidothymidine or Zidovudine (nucleoside reverse transcriptase inhibitors; NRTI), it became apparent that the virus is able to generate drug-resistant mutants [28,29]. Thus, looking at the high rates of virus production and mutation, ART regime now includes administration of two or more pharmacological agents in combination, referred to as highly active antiretroviral therapy (HAART) [27]. At present, more than 30 drugs have been approved by the United States Food and Drug Administration (FDA) for the treatment of HIV infection [30]. Currently, over 23.3 million HIV patients are receiving antiretroviral therapy across the globe [7]. 

### 2.1. Constraints to Current ART/HAART Regime

Antiretroviral drugs are suppressive, but cannot cure or eradicate the HIV-1 infection completely, i.e., the patients still carry HIV in their bodies in latent viral reservoirs, ready to rebound in case of HAART interruption [26,37,38]. The major drawbacks in the current antiretroviral medication are inadequate efficacy, incomplete coverage, unaffordability and compound toxicity. The complications arising from the emergence of resistant strains, further defies the therapeutic potential of these drugs. Moreover, along with various side effects, patients are at an increased risk of developing non-AIDS comorbidities, such as cardiovascular diseases, tuberculosis and cancers [39,40,41,42,43]. 

To control, and eventually eliminate, HIV globally, there is a need of widely accessible and efficient HIV prevention tools like vaccines. However, in the case of HIV, various attempts to develop a protective vaccine (for prevention) as well as a therapeutic vaccine (for treatment) have been severely compromised by the complex structure and nature of the virus. Additionally, the inability of the host to induce a sufficiently potent immune response against HIV is poorly understood [44]. Thus, there is currently no vaccine available that can prevent HIV infection or treat those who have it [45]. 

### 2.2. Preventive Measures

Advances are being made to develop prevention tools to protect people who are at substantial risk for HIV infection [46,47]. These are termed “pre-exposure prophylaxis (PrEP)” and “post-exposure prophylaxis (PEP)”, wherein taking a pill daily could provide protection against HIV. These include microbicides in the form of intravaginal rings and antiviral oral medications, etc. Moreover, after recognizing that taking a pill on a daily basis is uncomfortable and unachievable for some people, researchers are working to create new forms of PrEP that do not require a daily dose of HIV prevention medicines. There are different forms of long-acting PrEP that are being tested, such as implants releasing a fixed dose of antiretroviral drug over time, injectable long-acting antiviral agents and broadly neutralizing antibodies (bNAbs) [48]. On similar grounds, UNAIDS (Joint United Nations Programme on HIV/AIDS) has initiated LATITUDE (Long Acting Therapy to Improve Treatment sUccess in Daily lifE), a study which is comparing monthly injectable ART to daily oral drugs [49]. Whilst the search for an effective cure continues, the attempts to develop new drugs remain an important priority due to the development of resistance against existing drugs and the unwanted side effects associated with the current drugs.

## 3. Host–Viral Interactomes Offering New Drug Targets

Viruses are obligate intracellular parasites relying on host machinery for their survival. They are capable of engaging with numerous cellular proteins for successful viral replication. To achieve this, one of the ways virus use is the direct interaction of viral proteins with the host factors via binding sites. Alternatively, they have evolved intrinsically disordered, short protein regions called as eukaryotic linear motifs (ELMs) or short linear motifs (SLiMs) which mediate interactions with their host [50]. The mechanism often used by viruses is molecular mimicry, where ELMs acts as docking sites for several protein domains (e.g., SH3 and WW domains), as nuclear-localizing signal or for posttranslational modifications [51]. Although the set of identified ELMs is limited and studied only for a few viruses, it will be useful to comprehensively assess the contribution of ELMs in shaping the interaction network with the host. Undoubtedly, viruses have evolved a variety of strategies to manipulate the cellular machinery as well as to counteract or evade host immune defences.

### 3.1. Advances in Viral-Host Interactomics

Apparently, majority of viral replication steps are supported by interacting host proteins leading to the manipulation of cellular processes by viruses. Therefore, the interpretation of these physical interactions between viral and host proteins (V-H interactome) allows the identification of cellular proteins/functions that are essential in the virus life-cycle and thereby can be considered as new antiviral targets [52]. The study of V-H protein-protein interactions (PPI) data has been accelerated with time by the high-throughput screening techniques like Y2H (Yeast 2 Hybrid) system, protein array, protein complementation assays and co-affinity purification/MS [53]. We now have access to nearly complete interactomes for various viruses like influenza virus, hepatitis C virus (HCV) and dengue virus [54,55,56]. Furthermore, combining the evolving VH PPI information with the druggable interactions revealed a very large potential of drug repurposing for the discovery of molecules with antiviral activity [57]. 

### 3.2. Drug Discovery Now Targets Host Factors

**Figure 2:**
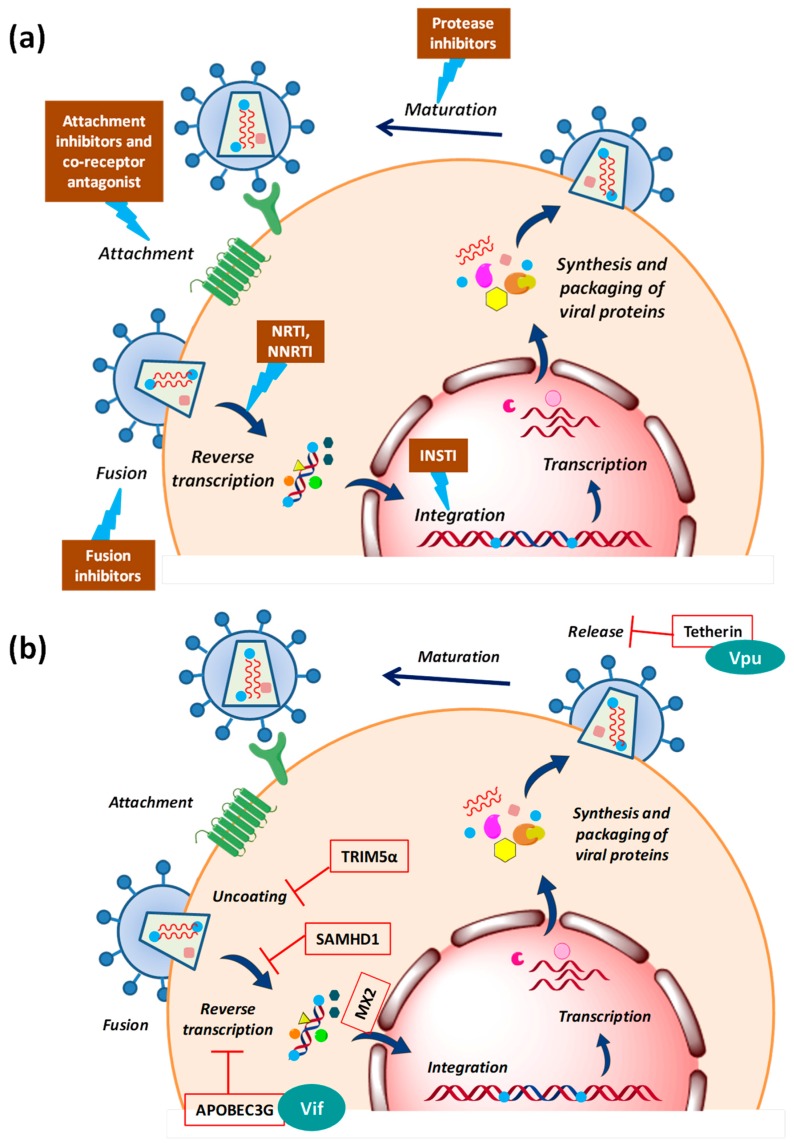
Diagrammatic representation of the sites of action of (**a**) clinical inhibitors of HIV-1 (ART) and (**b**) host restriction factors which are counteracted by HIV-1 accessory proteins. Abbreviations: APOBEC3G—apolipoprotein B mRNA editing enzyme, catalytic polypeptide-like 3G, TRIM5α—tripartite motif-containing protein 5α-isoform, SAMHD1—SAM domain and HD domain-containing protein 1, MX2—human myxovirus resistance 2, Vif—viral infectivity factor, Vpu—viral protein U.

The conventional antiviral (anti-HIV) drugs mainly target the three viral enzymes: Reverse transcriptase, integrase and protease, as shown in Figure 2a. Since the inventory of viral proteins which can be targeted for drug discovery is quite limited, a major drawback in the use of these direct-acting drugs is the emergence of resistance. This limitation has opened a new avenue in the antiviral drug discovery which now focuses exploring host-oriented molecules that are essential for viruses to replicate [58]. Promising results have been obtained in case of LASAG (d,l-lysine-acetylsalicylate glycine) against the Influenza virus. This compound with new antiviral mode of action inhibits signal transduction module which is essential for viral replication, has successfully completed Phase II clinical trials [59]. Another successful example is the development of co-receptor inhibitor maraviroc which blocks the cellular entry of HIV [60]. This is the first and only approved drug which targets the host protein. These encouraging results demonstrated the potential of cellular factors as drug targets, widening the repertoire of druggable molecules and also offer a greater barrier to the emergence of resistance. 

## 4. HIV-1-Host Interactors: Evaluating the Therapeutic Potential

### 4.1. Host Restriction Factors

During an HIV attack, the host cell does not offer a friendly environment to the virus. HIV faces several host defence factors which interfere with retroviral replication at different steps [61,62,63]. These restriction factors are the part of host’s innate anti-viral immunity that has possibly evolved under positive selective pressure due to past encounters with ancient viruses (Figure 2b). The first factor identified as the natural inhibitor of HIV-1 infection was a cytidine deaminase, APOBEC3G (apolipoprotein B mRNA editing enzyme, catalytic polypeptide-like 3G) which induces lethal hypermutations (deamination of C to U) in the HIV-1 genome, which are detrimental to viral replication [64,65]. Another restriction factor is Fv1/TRIM5α (tripartite motif-containing protein 5α-isoform), the C-terminus of which interacts directly with the incoming viral capsid causing premature uncoating of HIV-1 nucleocapsid, which is ultimately targeted to proteasomal degradation [66]. Tetherin/CD317 is another potent barrier to HIV-1 release that tethers the budding virions to the cell surface in order to prevent their efficient release and further triggers the proinflammatory signalling upon sensing the virion assembly [67]. HIV-1 replication is very inefficient in cells of the myeloid lineage, like dendritic cells. This could be because the host restriction factor SAMHD1 (SAM domain and HD domain-containing protein 1) regulates the dNTP pool in myeloid cells, thus blocking HIV reverse transcription by controlling the dNTP supply [68]. The most recently identified restriction factor is MX2/MXB (human myxovirus resistance 2) which is an interferon-induced protein. The N-terminal of MX2 containing a triple-arginine motif binds to HIV-1 capsid and blocks the nuclear import of viral cDNA [69]. 

These antiviral defence mechanisms would have been quite successful in the past encounters; however, HIV-1 has evolved the ability to counteract these by several accessory viral proteins (Figure 2b) like viral infectivity factor (Vif), viral protein U (Vpu)) or can evade them by its high variability and thus is capable to replicate efficiently even in the hostile environment of the host cell [70,71,72]. 

### 4.2. Druggable Host Factors Involved during HIV-1 Entry

The initial steps of HIV-1 infection have been studied in substantial detail with a large number of attempts made to block the well-defined interactions occurring during the HIV-1 entry. The first step of HIV-1 infection is the entry of virus which takes place in three sub-steps. First being the ‘attachment’ to the cell surface when gp120 (viral envelope protein) engages with a principal cell surface CD4 receptor. This interaction results in exposure of a ‘co-receptor binding’ site which interacts with an auxiliary co-receptor CCR5 or CXCR4. Subsequently ‘fusion’ occurs where gp41 exposes the fusion peptide to destabilize the host cell membrane and drives membrane fusion [73]. HIV-1 entry inhibitors, therefore, fall under three categories: attachment inhibitors, co-receptor binding inhibitors and fusion inhibitors. 

A variety of strategies have been pursued to develop entry inhibitors [73,74]. Attachment inhibitors include CD4 mimics, small molecule inhibitors which target CD4-gp120 binding site as well as CD4 antibodies (TNX-355). However, the oral bioavailability of the promising candidate was poor. To target the fusion step, peptide fusion inhibitors were designed which soon showed resistance compromising the drug efficiency. For inhibiting co-receptor binding, several modified forms of the natural ligand (CCL5/RANTES) of CCR5 were developed which were highly effective in vitro though failed in clinical trials [74,75]. Currently, there are no FDA approved attachment and fusion inhibitors in clinic.

Another strategy for co-receptor binding inhibition which worked well was the development of small molecules that bind to a hydrophobic pocket in CCR5 inducing conformational changes rather than directly occupying the gp120 binding site [74]. One of these inhibitors, maraviroc, has been approved by the US FDA and is currently the only antiretroviral drug targeting a cellular factor used in the clinic [60]. Several other related agents and anti-CCR5 antibodies are currently being evaluated in clinical trials [73,76] (see Box 1). 

### 4.3. Other HIV-1-Cellular Interactors as Possible Targets

HIV-1 can be tackled and/or inhibited at each step of its lifecycle in order to block its spread. Post-entry steps like uncoating, reverse transcription, nuclear entry, integration, transcription, nuclear exit, maturation, assembly and release from the cell membrane, all represent valid targets and are currently being investigated. For instance, during integration, disruption of the interaction between HIV-1 IN (integrase) and LEDGF/p75 (*l*ens epithelium-*d*erived *g*rowth *f*actor) leads to impaired viral replication. Targeting the interaction interface, thus, offers hope for a new target. Moreover, post-integration, different viral accessory proteins interact with cellular factors to achieve successful replication. For instance, during elongation of viral transcripts, HIV-1 Tat binds to transactivation response element (TAR, located in the HIV 5′-LTR) which in turn interacts with host factor p-TEFb (*p*ositive *t*ranscription *e*longation *f*actor *b*). So, Tat/TAR/P-TEFb interaction seems to be a promising target [77,78]. Similarly, targeting of DEAD-box helicases may inhibit HIV replication by suppressing function of Rev [79]. Furthermore, during virion release HIV-1 requires cellular ESCRT-I (endosomal sorting complex required for transport I) [80] Interactions between Gag and the endosomal sorting proteins Alix and TSG101 (tumor susceptibility gene 101) also suggest potential targets [81,82]. Based on these observations, various small compounds that disrupt such kind of host-virus interactions have been proposed and being tested. Moreover, since host factors involved are also necessary for the host cellular functions, designing an inhibitor against these has proven difficult and challenging.

Box 1 **The Berlin patient (2007) [83,84] and The London patient (2016) [85]: the only individuals ever to be cured of HIV/AIDS.**

Cure for HIV means elimination of all the infected cells from body. Standard ART is effective in reducing the viral load but cannot eradicate the viral reservoir from the patient’s body. The Berlin patient (Timothy Ray Brown) and the London patients shared similar medical circumstances. They both were HIV positive and receiving ART. Later, both developed cancer (acute myeloid leukemia and Hodgkin’s lymphoma, respectively) and required bone marrow transplants to replenish the stem cells which were destroyed by chemotherapy. Doctors in both instances used bone marrow cells from a CCR5 (Δ32/Δ32) donor. A homozygous mutation (Δ32/Δ32) in the CCR5 gene encoding the defective HIV co-receptor CCR5 confers resistance to HIV-1 infection. This Δ32/Δ32 allogenic stem cell transplantation in both the patients cured them with no viral detection for years and months. While these two cases have provided important insight for HIV research, bone marrow transplantation is a very risky procedure with a 50% chance of success and, hence, not a feasible strategy to cure HIV infection. The impetus obtained from these two cured patients led to the development of potent inhibitors of HIV co-receptors, but their clinical development was halted due to undesired side effects. Therefore, though the inhibition or knock-down of *CCR5* or *CXCR4* using small inhibitor molecules or gene therapy techniques is promising, there are some major concerns which need to be addressed carefully.

Diverse independent genome-wide RNA interference-based screens evaluated more than 842 genes that reduce HIV-1 infection when knocked-down [86,87,88]. Additionally, the National Library of Medicine has a comprehensive list of all cellular proteins shown to interact physically or functionally with HIV-1 available in Human Protein Interaction Database [89,90,91]. These screens provided a good starting point for the identification of cellular targets for HIV therapy. However, the cellular factors that overlapped in more than one screen were quite few. The important ones included host factors involved in HIV infection like surface/receptor molecules, cytoskeletal and signalling proteins, transcription and DNA binding proteins, translation and RNA binding proteins, etc. Some of the most interesting candidates belong to nucleoporins (Nups) and other NE (nuclear envelope)-associated proteins which are indispensable for the nuclear import and export of HIV.

## 5. Nuclear Entry and Egress of HIV-1

**Figure 3:**
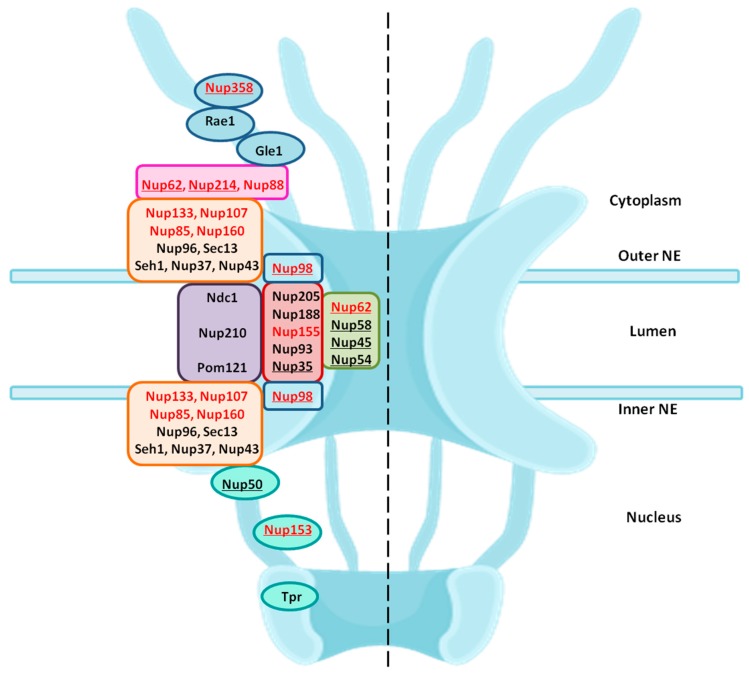
Structural organisation of nucleoporins in the NPC: Nups are organised into various subcomplexes, which here are shown in different coloured boxes. The location of Nups is marked on the left half of the NPC framework—the central transport channel/Nup62 subcomplex is shown in green; adapter ring/inner ring complex is in brick red; transmembrane Nups are in purple; Y-complexes are shown in orange; cytoplasmic ring Nups are in pink; Nups forming the cytoplasmic filaments are in light blue; nuclear basket is shown in teal color. The Nups highlighted in red are the ones identified as essential during the HIV-1 infection. The FG-Nups are underlined.

HIV is capable of infecting terminally differentiated and non-dividing cells [92]. The long interphase stage provides a very large benefit to HIV-1 and may account for its high replication rate observed. Entrance into and exit from the nucleus occurs via the nuclear pore complexes (NPCs) which are embedded in the double membrane of the nuclear envelope and mediate both passive (small molecules) and highly regulated active transport (cargoes larger than ~40 kDa) through the nuclear envelope. NPCs are macromolecular structures built from multiple copies of 30 different nucleoporins. These Nups are organized into various subcomplexes that are biochemically defined by their affinity to each other (Figure 3) [93,94,95,96]. One-third of all Nups are FG-Nups (Nup58, Nup54, Nup62, Nup45, Nup50, Nup98, Nup35, Nup214, and Nup358), containing phenylalanine-glycine repeats (FG-repeats). The FG Nups present at the central channel form a dense network of unstructured FG repeat regions which form the permeability barrier of the NPC and can transiently interact with soluble nuclear transport receptors (NTRs) conferring dynamic selectivity to the pore. NTRs like karyopherins (importins and exportins) recognize different types of nuclear localization signals (NLSs) or nuclear export signals (NESs) on cargo molecules and facilitate their import into or export from the nucleus, respectively [97,98,99]. 

During its whole lifecycle, HIV-1 encounters the nuclear membrane via NPC twice: (1) entry of HIV-1 PIC into the nucleus and (2) exit of viral RNAs after transcription into the cytoplasm. The dependency of HIV-1 on the nuclear envelope is reflected by the changes in the expression of nucleocytoplasmic shuttling proteins [100]. Various genome wide RNA interference (RNAi) screens and LC-MS/MS studies identified a list of NPC components and NE-associated proteins as essential host co-factors for HIV-1 infection. These include Nup98, Nup85, Nup133, Nup107, Nup160, Nup153, Nup214, Nup358, Nup155 and TNPO3 (tansportin 3) [86,87,100,101]. These Nups found in different screens belong to different subcomplexes in the NPC architecture. Some of these are FG-Nups which participate in nuclear transport. Figure 3 highlights the location of these Nups in their subcomplexes within the NPC. 

### 5.1. Nuclear Import of PIC

**Figure 4:**
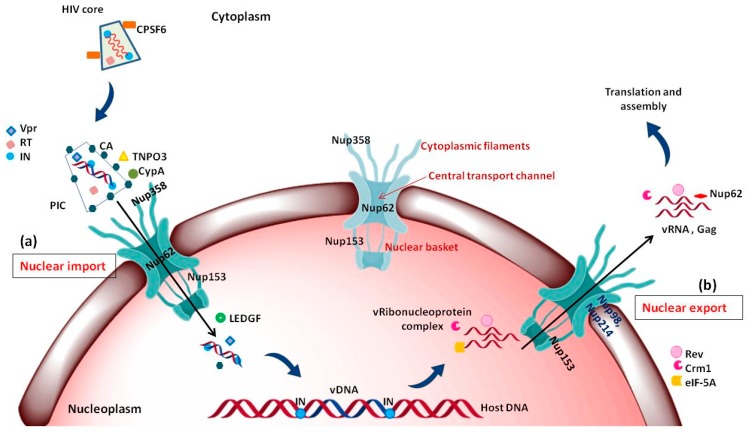
Major events occurring during nuclear import and export of HIV-1: (**a**) Nuclear import of HIV-1 PIC: CPSF6 interacts with intact HIV core which upon uncoating and reverse transcription forms the PIC. TNPO3, CypA and CypA like domain of Nup358 binds to the left over CA and helps in docking of PIC on the cytoplasmic face of NPC; while Vpr and IN having NLS, interact with Nups like Nup153 which help in import of the PIC into the nucleus. In nucleoplasm, LEDGF is involved in release of PIC from Nup153 and helps in integration of vDNA into the host DNA; (**b**) nuclear export of vRNA-protein complex: The unspliced and partially spliced mRNA need viral accessory protein Rev which interacts with various cellular factors like Crm1, eIF-5A and Nups, such as Nup62, Nup214, Nup98 and facilitate the nuclear export of this viral ribonucleoprotein complex. Abbreviations: NPC—nuclear pore complex, CPSF6—cleavage and polyadenylation specific factor 6, TNPO3—transportin 3, CypA—cyclophillin, CA—capsid, Vpr—viral protein r, IN—integrase, RT—reverse transcriptase, PIC—pre initiation complex, LEDGF—lens epithelium-derived growth factor, IN—integrase, Crm1—chromosome region maintenance- 1, Rev—regulator of virion expression, eIF-5A—eukaryotic translation initiation factor-5A.

Reverse transcription of the viral RNA genome into viral DNA takes place in the cytoplasm followed by translocation to the nucleus. The newly synthesized viral DNA remains associated with viral and cellular proteins in a large nucleoprotein complex called the pre-integration complex (PIC) [102,103,104]. HIV-1 PICs were shown to contain several NLS containing viral proteins such as matrix (MA), Integrase (IN) and viral protein R (Vpr) [105,106,107,108,109,110]. There are different Nups and nuclear factors that have the ability to bind to HIV-1 cores and to participate in the early steps of HIV-1 infection: TNPO3, CPSF6, Nup358/RanBP2, Nup98, Nup214 and Nup153 (Figure 4a) [111,112]. Upon depletion, all four Nups induced defects in HIV-1 infectivity. Nup153 was found to participate in nuclear import of HIV-1 PIC; while, Nup358 interacts with the HIV-1 core and mediate its docking at the nuclear pore [101]. Interestingly, knockdown of other two Nups: Nup98 and Nup214 lead to decreased HIV-1 infectivity but are not directly involved in nuclear import of HIV-1 PIC [101]. Another study further suggested direct interaction of Nup62 with the HIV-1, which might play a role in import and chromosome tethering [113]. Following are few examples highlighting the involvement of Nups in HIV-1 docking at the NPC and nuclear entry.

#### 5.1.1. Interaction of Nup358 with HIV-1 CA

**Figure 5:**
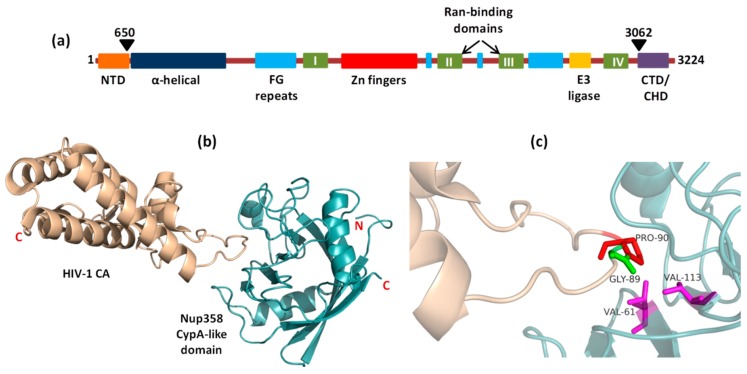
Complex of HIV-1 CA (wheat) and Nup358CypA (Cyan) (PDB code 4LQW): (**a**) Domain architecture of human Nup358 (**b**) Loop of HIV-1 CA bound in cleft of Nup358CypA; (**c**) Val61 and 113 which form the hydrophobic pocket in Nup358 CHD, interact with Gly89 and Pro90 of Cyp binding loop in HIV-1 CA.

Nup358 assist HIV-1 core to dock at the NPC via interaction with viral CA protein. Nup358/RanBP2 is the largest Nup situated in the cytoplasmic fibrils of NPC (Figure 3 and Figure 4a). It is a multidomain assembly (Figure 5a) containing a N-terminal α-helical region, four Ran-binding domains, eight zinc-finger motifs, E3 ligase domain and a C-terminal cyclophilin A (CypA) - homologous domain (CHD) and multiple FG-repeat domains which are involved in the binding and transport of cargo-receptor complexes through the NPC [114,115]. When the HIV-1 core enters the cell upon fusion of viral envelope with cellular membrane, disassembly of the core is initiated, releasing the RTC (*r*everse *t*ranscription *c*omplex) where reverse transcription of viral RNA takes place into cDNA. However, the precise location and mechanism of CA core uncoating remains controversial [12,116]. While the initial steps of core uncoating are linked to reverse transcription, subsequent events may involve binding to host proteins like CPSF6 and Cyp A (cyclophilin A) and a little leftover CA also remains associated with the HIV-1 PIC (Figure 4a) [117]. The CypA and the Nup358’s cyclophilin-homologous domain (CHD), both bind to the cyclophilin binding loop (with GP residues) exposed on the top of CA N- terminal domain (Figure 5b,c). When the structures of Nup358 CHD and CypA with CA were overlapped, the binding of both Nup358 CHD and CypA in the proline-rich loop of HIV-1 CA appeared similar [118]. Reports suggest that the peptidyl-propyl isomerase activity exhibited by Nup358 could destabilize the viral core by causing *cis*-to-*trans* isomerization which lead to conformational changes in the capsid and thereby promote uncoating [118,119,120,121]. It was further hypothesized that the interaction between the CHD of Nup358 and the capsid molecules associated with the HIV-1 PIC could dock the PIC to the cytoplasmic side of the NPC for translocation into the nucleus [101]. However, the lack of structural information about the full length Nup358 limits our knowledge regarding its role in regulating the cellular functions as well as during interaction with capsid protein.

#### 5.1.2. Interaction of Nup153 with Different HIV-1 Proteins

**Figure 6:**
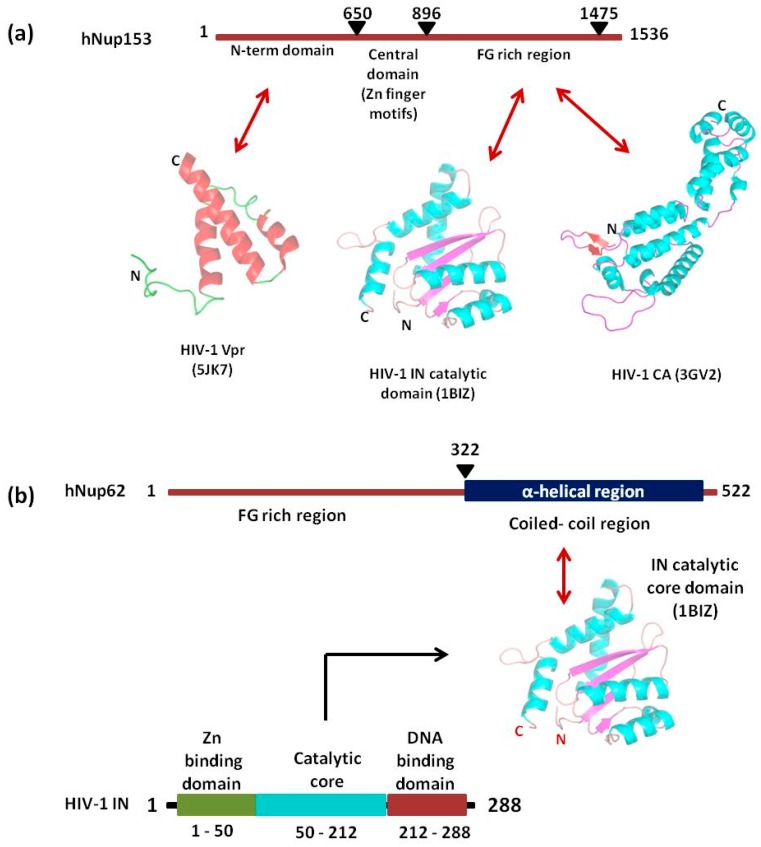
Schematic representation of interacting regions of Nup153 (**a**) and Nup62 (**b**) with different HIV-1 proteins.

Nup153 is a nucleoplasmically oriented Nup that predominantly located to the nuclear side of the NPC as a member of nuclear basket (Figure 3). While the N-terminal domain of Nup153 is anchored to the nuclear rim of the NPC, its C-terminal FG rich domain is natively unfolded with no appreciable secondary structure. It has been shown that the 200 nm long and flexible Nup153 C-terminal can reach to the cytoplasmic side of the NPC channel. Human Nup153 C-terminal contains 29 FG motifs (FxF, FG and FxFG patterns), which play a vital role in Nup153-mediated nucleocytoplasmic transport [122,123,124,125,126]. The dynamic Nup153 located in the nuclear basket of NPC plays a significant role in HIV-1 PIC import. It binds directly with HIV-1 IN, CA, and Vpr in an α-importin-dependent and (or) β-importin-independent manner for import. The contact between the C-terminal domain of Nup153 and the PIC is made at the cytoplasmic face of the NPC. One of the key event in the nuclear import of the HIV-1 PIC is the direct interaction between the C-terminal domain (amino acid residues 896–1475) of NUP153 and PIC-associated IN (Figure 6a) [127]. The same FG domain also binds to the PIC-associated CA molecules [128]. These interactions mediated by Nup153 help in drawing the PIC into the nuclear side through the NPC. Moreover, the viral protein Vpr has also been shown to interact with a region of Nup153 in the N-terminal domain (amino acids 447–634), but the mechanism and significance of this interaction is still not clear (Figure 6a) [129]. 

#### 5.1.3. Role of Nup62

Nup62, a glycosylated FG-Nup is a component of the central transport channel (CTC) of the NPC (Figure 3). This Nup62 sub complex (composed of Nup62 along with Nup58, Nup45 and Nup54) forms a dense network of unstructured FG repeat regions to regulate the nucleocytoplasmic transport via interactions between their FG domains and the karyopherins [95,97]. Nup62 consists of two distinct domains, the N-terminal FG-rich region and the C-terminal α-helical coiled-coil domain (Figure 6b). The C-terminal (amino acids 328–522) domain of Nup62 facilitates nucleocytoplasmic transport interacting with nuclear transport receptors and/or cargoes [95]. The coiled-coil domains present in the α-helical regions of Nup62 enables it to form homotrimer and heterotrimer either with Nup54 or Nup54-Nup58 within the CTC. This coiled coil motif is also responsible for various protein-protein interactions like with that of Exo70 (a member of the exocyst complex in the cytosol) [130]. Furthermore, in the context of evolution of the CTC nups, Nup62 is comparatively conserved from fungi to metazoan [131]. Nup62 not only contributes in nuclear transport but is also involved at several steps during HIV-1 replication. The coiled-coil domain of Nup62 is shown to interact directly with HIV-1 IN and aid in viral integration (Figure 6b) [113]. The level of viral-integrated DNA in Nup62-knockdown cells was drastically decreased indicating a major effect of Nup62 on integration step [113]. Possibly, by interacting with HIV-IN, Nup62 offers the HIV PIC a direct entry way from the NPC to the chromosomal DNA, thus enabling efficient integration. Additionally, immunofluorescence and co-localization studies suggested that Nup62 is essential for viral genomic RNA (vRNA) export and revealed that Nup62 is exported from the nucleus along with Rev, vRNA and Gag into the cytoplasm (Figure 4b) [100]. In normal cells, Nup62 resides mostly in the nuclear envelope; while, during HIV-1 infection, it is re-distributed to the nucleoplasm and then to cytoplasm, where it resides with the viral ribonucleoprotein complex [100]. 

It is noteworthy that NPC components not only facilitate nuclear import of the PIC but also plays a role during the integration of the proviral cDNA into the host chromosome. Few Nups, particularly Nup62, Nup153 and Nup98, can be found in the nucleoplasm and regulate the expression of certain cellular genes [132]. These Nups might be contributing in HIV-1 infection by interacting with HIV-IN and cellular factor LEDGF in the nucleoplasm and locating the integration site in the host chromosome followed by successful proviral integration [113,132,133]. Thus, various Nups are found to be involved directly or indirectly in HIV-1 replication at different steps.

### 5.2. Nuclear Export of Viral mRNAs

Upon integration of the vDNA into the host chromosome, the viral 5′-LTR acts as a weak promoter for the transcription of viral mRNA. The viral mRNA is spliced using host splicing mechanisms and results in three different mature RNAs, viz. completely spliced, partially spliced and unspliced RNAs. The latter two are too big to be exported into the cytoplasm. Hence, only the spliced vRNA expressing the viral proteins Rev, Tat and Nef are readily translocated to cytoplasm [134,135]. The expressed Tat and Rev proteins possess arginine-rich NLS which helps in translocating them to the nucleus via interaction with importin β [136]. In the nucleus, Tat transactivates the 5′-LTR, increasing the level of viral mRNA transcription and thereby protein expression [137]. The Rev protein promotes the nuclear export of unspliced (act as viral genome) and partially spliced (encoding gag proteins) forms of viral mRNA to the cytoplasm [17,138]. The import of Rev is also aided by another host protein namely, nucleolar phosphoprotein B23 [139]. Rev also has a leucine-rich NES and interacts with a number of cellular proteins like Crm1, eIF-5A and Nups in order to facilitate this export of Rev-RRE RNA complex. eIF-5A localized at the nucleoplasmic face of the NPC interacts with Nup214, Nup153, Nup98 and Nup62, which are involved in nuclear export [140,141,142,143,144]. eIF-5A may act as an adapter that targets the Rev-NES to the nucleoplasmic face of the NPC and mediates efficient binding to Crm1. Interestingly, Nup62 translocates to cytoplasm along with viral Gag, RNAs and Rev (Figure 4b) [100]. The immunoblot analysis of purified virions identifies Nup62 as an incorporated, virion-associated protein and suggests that Nup62 may be important for HIV-1 assembly [100]. In summary, Nups also are associated with the export of viral ribonucleoprotein complex into the cytoplasm; however, the mechanism of this export is not clearly understood.

Overall, HIV-1 utilizes components of the NPC for both PIC import and Rev-vRNA RNP export. Hence, inhibiting the interactions between HIV-1 proteins and Nups might be helpful in developing novel anti-viral strategies. Figure 6 summarizes the interactions between HIV-1 and host nuclear factors including Nups and karyopherins which are important for HIV-1 replication. The interactome comprises of various host–viral interactions occurring during nuclear import and export steps in the life cycle of HIV-1. The four viral proteins playing a crucial role in these steps are CA, Vpr, IN and Rev. While the Nups and associated nuclear proteins which contribute maximally in these interactions are Nup358, Nup153, Nup62, Nup214, Nup98, TNPO3, LEDGF, Impα and Crm1.

## 6. Significance of Targeting Nup-HIV-1 Interactions as Drug Targets 

The nuclear pore complex plays a significant role as HIV-1 host partner and helps in the nuclear translocation of viral DNA, RNA and proteins. Nups are the major components of the NPC which not only contribute to NPC assembly and overall architecture and but also maintains a strict permeability barrier by allowing selective active translocation of macromolecules through the NPC. Additionally, Nups are also involved in the regulation of cellular gene expression and interaction with chromatin in the nucleoplasm [145]. Any dysregulation of the nucleocytoplasmic transport is associated with the pathogenesis of various diseases, such as cancer, viral infections and neurodegenerative conditions [146,147,148]. Different studies have shown that the NPCs are heavily remodelled in cancer as well as in HIV-1 infection. Employing LC-MS/MS studies, Chan et al. [149,150] and Monette et al. [100] have independently highlighted the upregulation and downregulation of NPC and shuttling proteins by HIV-1. Since Nups prove to be an essential requirement for the nuclear entry of the PIC, the nuclear export of the vRNA RNP, and thereby for virus infectivity, the prospect of targeting the interacting protein domains of Nups and viral components may be targeted to bring the HIV-1 replication cycle to a halt.

A number of reports have also pointed towards the compositional variability of the NPC both within and between the cells [151,152]. Further, an altered expression under certain disease conditions has been reported [100,146]. These reports raise the fascinating opportunity that NPCs with different compositions may possess different functional properties in different cells. It is now evident that NPCs are highly dynamic complexes with many transport-independent functions and that the expression of several NPC components vary among different cell types and tissues and also during development [153,154]. These findings, together with the fact that mutations in certain Nups result in tissue-specific diseases, give an impression that NPC composition may play an important role in cellular function. The NPC variability is observed not only in HIV-1 infected and uninfected cells but also in immune cells and other body cells. This cell- and tissue-specific compositional variation of Nups presents another prospect which should be thoroughly understood in order to harness their potential.

**Figure 7:**
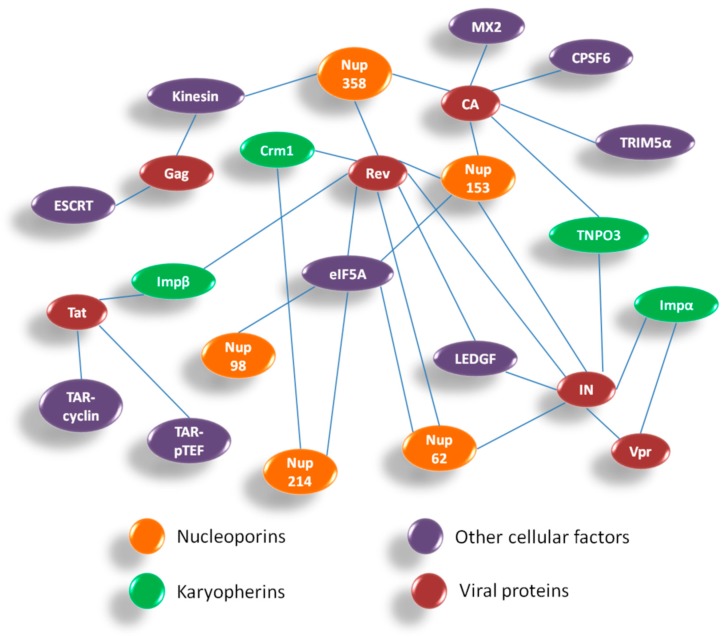
Host-HIV-1 interactome during nuclear import and export of HIV-1 at a glance. Abbreviations: ESCRT—endosomal sorting complex required for transport, TAR—transactivation response element, pTEFb—positive transcription elongation factor b, Imp—importin, Crm1—chromosome region maintenance-1, eIF5A—eukaryotic translation initiation factor-5A, CA—capsid; MX2—human myxovirus resistance 2; CPSF6—cleavage and polyadenylation specific factor 6, TRIM5α—tripartite motif-containing protein 5α-isoform, TNPO3—transportin 3, IN—integrase, Vpr—viral protein r, Tat—transactivator of transmembrane, Rev—regulator of virion expression.

The physiological and pathophysiological significance of the nuclear membrane and nucleocytoplasmic transport has been recently reported [155,156]. Treating NTRs as the therapeutic targets against various diseases has led to the development of nuclear transport inhibitors [156]. Leptomycin B (LMB) which covalently binds to C-528 in the central conserved binding region of Crm1 blocks its interaction with the NES of a cargo protein in an irreversible manner [157,158]. LMB was tested in phase-I clinical trials to treat advanced refractory cancer; however, the clinical trials were discontinued due to significant systemic toxicity [159]. Similarly, there were more than 20 nuclear transport inhibitors described of which very few could be monitored in a clinical setting [160]. Due to the indispensable role of nuclear import and export pathways in normal cell functioning, most of the broad-spectrum inhibitors were unsuccessful. The inhibitors which can specifically target the Nup–HIV-1 interaction interfaces can be viewed as an innovative class of therapeutic intervention. Some small molecules and short peptides have been designed which could modulate such interactions. These act either by specifically interacting with the NLS domain of a karyophilic protein and thus preventing its interaction with the appropriate importin, or they can mimic the domain which competes with the NLS carrying protein for interaction with importin [156]. Examples include peptides blocking HIV-1 IN interaction with importin α and LEDGF [161,162]. On similar grounds, host Nups - HIV-1 interactions like Nup153 with IN or CA, Nup358 with CA, etc., can be targeted to develop and test new antiviral compounds. However, one must keep in mind that some level of cytotoxicity is always associated while considering any cellular protein. Nonetheless, it can now be expected that new findings will enrich the existing knowledge about the role of Nups and NE-associated proteins in HIV-1 infection. This would provide the design of compounds targeting not only viral or cellular proteins, but their ‘interactions’ which might pose less toxicity and low chances of drug resistance (Figure 7). Therefore, specific inhibitors for HIV interactions with Nups and NTR, alone or in combination with other antiviral agents might be a promising strategy to target HIV infection.

## 7. Challenges and Perspectives

**Figure 8:**
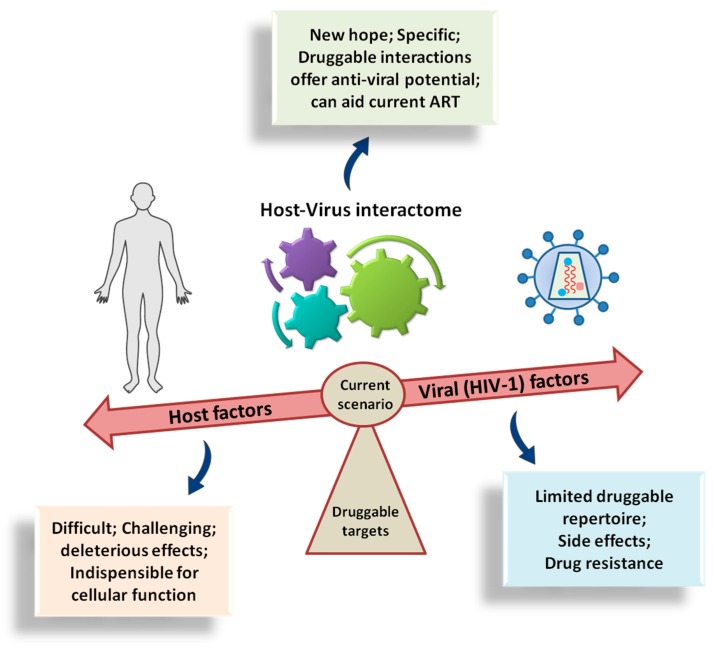
Cartoon depiction of the current scenario showing the drug targets for anti-viral (anti-HIV) medication.

HIV still remains a major global concern. Although there is no sterilising cure or preventive vaccine at present, a functional cure for HIV-1 might be more realistic to achieve. To date, all the antiviral drugs in clinical use for HIV-1 have been designed either to inhibit one of the three viral enzymes or to block the viral entry into the cell during attachment and fusion with the cellular membrane. The ART have certainly resulted in an appreciable decrease in HIV infections. However, development of HIV-1 resistance to these drugs has become one of the greatest challenges in identifying an appropriate combination of drug regimes with the fewest side effects for the treatment of patients. Figure 8 summarises the current scenario of the drug targets for developing anti-HIV medication. Consequently, there is a clear need to identify and develop new targets and novel drugs for the treatment of HIV-1 infection. The two areas of scope are presented by targeting the HIV-1 regulatory proteins, such as Tat and Rev, and by strengthening the host restriction factors like APOBEC3G, etc. Another direction is offered by the nuclear transport factors including NTRs and Nups, both of which are important for HIV replication and are yet to be exploited for clinical treatment of HIV-1-infected patients. Development of new drugs that consider these proteins would considerably increase the number of available treatment options for HIV-1. 

Given that the interaction between HIV-1 and its human host is far more complex than previously anticipated, a major challenge to the field will be the identification of essential interactions between viral and host factors which can be blocked without important physiological side-effects/consequences. Moreover, the drugability of the identified potential targets poses another serious challenge. In the past decades, structure-guided drug discovery has led to the development of various molecules which are being tested for clinical trials. The structural information available for HIV-1 is far more than any other virus; yet we are limited by the structural knowledge of host-viral protein complexes in both infected and uninfected conditions. The in-depth understanding of the structural and functional aspects of cellular proteins, such as Nups and other nuclear factors, which are important in HIV-1 life cycle is needed, which can then be exploited to speculate disease conditions. Even though a large number of cellular protein-protein interactions have been described, the way to the development of clinical drugs is still far. Nevertheless, significant progress is being made in the field and new targets are being discovered to keep the journey of developing novel antivirals going and as well as keep the hope to win over HIV/AIDS alive.

## Figures and Tables

**Table 1:** HIV-encoded proteins and their functions.

Class	Protein	Function
Viral enzymes (encoded by *pol*)	Reverse transcriptase (RT)	builds a DNA copy of the viral RNA genome; reverse transcription and RNAse H activity [9]
	Integrase (IN)	takes the DNA copy of the viral genome inside nucleus and inserts it into the infected cellular genome [10]
	Protease (PR)	essential for cleavage of polypeptides and maturation of HIV particles [11]
Structural proteins (encoded by *gag* and *env*)	Matrix (MA)	forms a coat on the inner surface of the viral membrane [12]
	Capsid (CA)	forms a cone-shaped coat around the viral RNA, delivering it into the cell during infection [13]
	Nucleocapsid (NC)	forms a stable complex with the viral RNA to protect it [14]
	Gp120 (SU envelope protein)	bind to receptors on the surface of host cell and then penetrate the cell surface making way for fusion [15,16]
	Gp41 (TM envelope protein)	
Accessory proteins	Tat	transactivator of transmembrane, enhances transcription of viral mRNA [17]
	Rev (regulator of virion expression)	regulates the splicing and nuclear export of viral RNA [18]
	Nef (negative regulatory factor)	inhibits host’s defences; pleiotropic effects, can increase or decrease virus replication [19]
	P6	incorporation of Vpr into new viruses; virion budding [20]
	Vif (viral infectivity factor)	increases virus infectivity by inhibiting host’s defence proteins; helps in virion assembly [21]
	Vpr (viral protein r)	nuclear import of viral DNA [22,23]
	Vpu (viral protein u)	Helps in virus budding and release [24]

**Table 2:** Types of antiviral drugs for HIV treatments.

Drug Classes	Mode of Action
Receptor/co-receptor antagonist	Inhibits HIV fusion to host cells (gp120/CCR5/CXCR4) [31]
Fusion Inhibitors	Blocks virus penetration through host cell membrane [32]
NNRTI	Non-nucleoside reverse transcriptase inhibitors; binds at position distant from active sites of RT [33]
NRTI	Nucleoside reverse transcriptase inhibitors; competitive inhibitor [34]
PR inhibitors	Inhibits protease; prevents virion maturation [35]
INSTI	Integrase strand transfer inhibitor; prevents vDNA integration into the host genome [36]

* Table modified from reference [10].

## Abbreviations

AIDS	Acquired Immunodeficiency Syndrome
APOBEC3G	Apolipoprotein B mRNA Editing Enzyme Catalytic polypeptide-like 3G
CA	Capsid
CHD	Cypa Homology Domain
CPSF6	Cleavage and Polyadenylation Specific Factor 6
CypA	Cyclophillin A
eIF-5A	Eukaryotic translation Initiation Factor-5A
ESCRT-I	Endosomal Sorting Complex Required for Transport I
FDA	Food and Drug Association
HAART	Highly Advanced AntiRetroviral Therapy
HIV	Human Immunodeficiency Virus
IN	Integrase
Crm1	Chromosome Region Maintenance-1
LEDGF	Lens Epithelium Derived Growth Factor
LTR	Long Terminal Repeat
MX2	human Myxovirus resistance 2
NPC	Nuclear Pore Complex
Nup	Nucleoporin
NTR	Nuclear Transport Receptor
NLS	Nuclear Localisation Signal
NES	Nuclear Export Signal
PIC	Pre Initiation Complex
PPI	Protein-Protein Interaction
pTEFb	positive Transcription Elongation Factor b
Rev	regulator of virion expression
RT	Reverse Transcriptase
SAMHD1	SAM domain and HD domain-containing protein 1
TNPO3	transportin 3
Tat	Transactivator of Transmembrane
TRIM5α	Tripartite Motif-containing protein 5α- isoform
TSG101	Tumor Susceptibility Gene 101
UNAIDS	joint United Nations program for Acquired Immunodeficiency Syndrome
V-H interactome	Viral Host Interactome
Vif	Viral Infectivity Factor
Vpr	Viral Protein R
Vpu	Viral Protein U
